# Supplementary material for: Long-Term Outcomes of in Utero Ramadan Exposure: A Systematic Literature Review
Source: Nutrients. 2021 Dec 17;13(12):4511. doi: 10.3390/nu13124511 (PMC8704584; doi:10.3390/nu13124511)
Supplement: Supplementary file 1 [file nutrients-13-04511-s001.zip › nutrients-1509874-supplementary.pdf]

SUPPLEMENTARY MATERIALS

Long-term outcomes of in utero Ramadan exposure: a systematic literature review

Melani R. Mahanani, Eman Abderbwih, Amanda S. Wendt, Andreas Deckert, Khatia Antia,

Olaf Horstick, Peter Dambach, Stefan Kohler, Volker Winkler

Table S1. Full search strategy for all databases 2

Table S2. Quality assessment of included studies using the Specialist Unit for Review Evidence (SURE) checklist. 3

Table S3. PRISMA Checklist........ 5

Table S1. Full search strategy for all databases
Database last searched on 21 November 2021

Source (date)	Search String	Hits	Hits after deduplication
PubMed (21.11.2021)	(ramadan* NOT ramadan*[author]) OR (ramadan*[Title/Abstract] AND ramadan*[Author])	1737	
Web of Science (21.11.2021)	(ALL=ramadan* NOT AU=ramadan*) OR (TS=ramadan* AND AU=ramadan*)	3954	
EconLit, PsycINFO, and Index Islamicus (through EbscoHOST) (21.11.2021)	(TX Ramadan* NOT AU Ramadan*) OR (TI Ramadan* AND AU Ramadan*)	1075	
Cochrane Database of systematic reviews (21.11.2021)	(Ramadan*) NOT (Ramadan*):au OR (Ramadan*):ti,ab,kw AND (Ramadan*):au	9	
WHO Global Index Medicus (21.11.2021)	tw:(ramadan*)	347	
WHO Virtual Health Library (21.11.2021)	tw:(ramadan*)	1568	
Total		8690	8304

Source (date)	Search String	Results
Google Scholar (21.11.2021)	Ramadan	474,000

Table S2. Quality assessment of included studies using the Specialist Unit for Review Evidence (SURE) checklist. Continued.

**Authors, year**	**1. Is the study design clearly stated? (yes/no/CD)**	**2. Does the study address a clearly focused question?**	**3. Are the setting, locations and relevant dates provided?**	**4. Were participants fairly selected?**	**5. Are participant characteristics provided?**	**6. Are the measures of exposures & outcomes appropriate?**	**7. Was bias considered?**
Pradella and van Ewijk, 2018	yes	yes	yes	yes	yes	yes	yes
van Ewijk et al, 2013	no	yes	yes	yes	yes	yes	yes
van Ewijk, 2011	no	yes	yes	yes	no	yes	yes
Majid et al, 2019	yes	yes	yes	yes	yes	yes	yes
Kunto and Mandemakers, 2019	no	yes	yes	yes	yes	yes	yes
Majid, 2015	yes	yes	yes	yes	yes	yes	yes
Chaudhry and Mir, 2021	yes	yes	yes	yes	yes	yes	yes
Azizi et al, 2004	yes	yes	yes	yes	no	yes	no
Karimi et al, 2021	no	yes	yes	yes	yes	yes	yes
Almond et al, 2011	yes	yes	yes	yes	no	yes	yes
Greve et al, 2017	yes	yes	yes	yes	no	yes	yes
Schultz-Nielsen et al, 2016	yes	yes	yes	yes	yes	yes	no
Schoeps et al, 2018	yes	yes	yes	yes	yes	yes	yes
Lee et al, 2020	yes	yes	yes	yes	yes	yes	yes
Almond and Mazumder, 2011	yes	no	yes	yes	yes	yes	yes
Karimi and Basu, 2018	no	yes	yes	yes	yes	yes	no

Authors, year	8. Is there a description of how the study size was arrived at?	9. Are the statistical methods well described?	10. Is information provided on participant flow?	11. Are the results well described?	12. Is any sponsorship/conflict of interest reported?	13. Finally…Did the authors identify any limitations and, if so, are they captured above?	Total
Pradella and van Ewijk, 2018	no	yes	no	yes	yes	yes	11
van Ewijk et al, 2013	no	yes	yes	yes	yes	yes	11
van Ewijk, 2011	no	yes	yes	yes	no	no	8
Majid et al, 2019	no	yes	yes	yes	yes	yes	12
Kunto and Mandemakers, 2019	no	yes	yes	yes	yes	yes	11
Majid, 2015	no	yes	no	yes	no	no	9
Chaudhry and Mir, 2021	no	yes	yes	yes	yes	yes	12
Azizi et al, 2004	yes	yes	no	yes	no	no	8
Karimi et al, 2021	no	yes	yes	yes	no	yes	10
Almond et al, 2011	no	yes	No	yes	no	yes	9
Greve et al, 2017	no	yes	yes	yes	no	no	9
Schultz-Nielsen et al, 2016	no	yes	no	yes	no	yes	9
Schoeps et al, 2018	no	yes	yes	yes	yes	yes	12
Lee et al, 2020	no	yes	no	yes	yes	yes	11
Almond and Mazumder, 2011	no	yes	no	yes	no	yes	9
Karimi and Basu, 2018	no	yes	yes	yes	no	yes	9

Table S3. PRISMA Checklist

Section/topic	#	Checklist item	Reported on page #
**TITLE**			
Title	1	Identify the report as a systematic review, meta-analysis, or both.	1
**ABSTRACT**			
Structured summary	2	Provide a structured summary including, as applicable: background; objectives; data sources; study eligibility criteria, participants, and interventions; study appraisal and synthesis methods; results; limitations; conclusions and implications of key findings; systematic review registration number.	1
**INTRODUCTION**			
Rationale	3	Describe the rationale for the review in the context of what is already known.	1,2
Objectives	4	Provide an explicit statement of questions being addressed with reference to participants, interventions, comparisons, outcomes, and study design (PICOS).	2
**METHODS**			
Protocol and registration	5	Indicate if a review protocol exists, if and where it can be accessed (e.g., Web address), and, if available, provide registration information including registration number.	-
Eligibility criteria	6	Specify study characteristics (e.g., PICOS, length of follow-up) and report characteristics (e.g., years considered, language, publication status) used as criteria for eligibility, giving rationale.	2,3
Information sources	7	Describe all information sources (e.g., databases with dates of coverage, contact with study authors to identify additional studies) in the search and date last searched.	2,3
Search	8	Present full electronic search strategy for at least one database, including any limits used, such that it could be repeated.	Table S1
Study selection	9	State the process for selecting studies (i.e., screening, eligibility, included in systematic review, and, if applicable, included in the meta-analysis).	2,3

Data collection process	10	Describe method of data extraction from reports (e.g., piloted forms, independently, in duplicate) and any processes for obtaining and confirming data from investigators.	2,3
Data items	11	List and define all variables for which data were sought (e.g., PICOS, funding sources) and any assumptions and simplifications made.	2,3
Risk of bias in individual studies	12	Describe methods used for assessing risk of bias of individual studies (including specification of whether this was done at the study or outcome level), and how this information is to be used in any data synthesis.	2,3
Summary measures	13	State the principal summary measures (e.g., risk ratio, difference in means).	-
Synthesis of results	14	Describe the methods of handling data and combining results of studies, if done, including measures of consistency (e.g., I^2) for each meta-analysis.	-
Risk of bias across studies	15	Specify any assessment of risk of bias that may affect the cumulative evidence (e.g., publication bias, selective reporting within studies).	-
Additional analyses	16	Describe methods of additional analyses (e.g., sensitivity or subgroup analyses, meta-regression), if done, indicating which were pre-specified.	-
**RESULTS**			
Study selection	17	Give numbers of studies screened, assessed for eligibility, and included in the review, with reasons for exclusions at each stage, ideally with a flow diagram.	3
Study characteristics	18	For each study, present characteristics for which data were extracted (e.g., study size, PICOS, follow-up period) and provide the citations.	3-6
Risk of bias within studies	19	Present data on risk of bias of each study and, if available, any outcome level assessment (see item 12).	6, Table S2
Results of individual studies	20	For all outcomes considered (benefits or harms), present, for each study: (a) simple summary data for each intervention group (b) effect estimates and confidence intervals, ideally with a forest plot.	6-8
Synthesis of results	21	Present results of each meta-analysis done, including confidence intervals and measures of consistency.	-
Risk of bias across studies	22	Present results of any assessment of risk of bias across studies (see Item 15).	-
Additional analysis	23	Give results of additional analyses, if done (e.g., sensitivity or subgroup analyses, meta-regression [see Item 16]).	-
**DISCUSSION**			
Summary of evidence	24	Summarize the main findings including the strength of evidence for each main outcome; consider their relevance to key groups (e.g., healthcare providers, users, and policy makers).	9

Limitations	25	Discuss limitations at study and outcome level (e.g., risk of bias), and at review-level (e.g., incomplete retrieval of identified research, reporting bias).	9,10
Conclusions	26	Provide a general interpretation of the results in the context of other evidence, and implications for future research.	10
**FUNDING**			
Funding	27	Describe sources of funding for the systematic review and other support (e.g., supply of data); role of funders for the systematic review.	10

From: Moher D, Liberati A, Tetzlaff J, Altman DG, The PRISMA Group (2009). Preferred Reporting Items for Systematic Reviews and Meta-Analyses: The PRISMA Statement. PLoS Med 6(7): e1000097. doi:10.1371/journal.pmed1000097

For more information, visit: **www.prisma-statement.org**.
